# Conservatively Treated Giant‐Sized Rasmussen Aneurysm: Case Report of a Rare Sequela of Pulmonary Tuberculosis

**DOI:** 10.1002/ccr3.9718

**Published:** 2024-12-10

**Authors:** Prosper Adjei, Edward Ebo Ocran, Jerry Kevin Boafo Osei‐Alomele, Belynda Abiti

**Affiliations:** ^1^ Department of Internal Medicine Methodist Hospital Wenchi Ghana; ^2^ Department of Radiology Holy Family Hospital Techiman Ghana

**Keywords:** hemoptysis, lung cavity, pulmonary artery pseudoaneurysm, pulmonary tuberculosis, Rasmussen aneurysm

## Abstract

Rasmussen aneurysm is a rare cause of tuberculosis‐related hemoptysis, which affects about 0.007% of the general population. This case report highlights the role of conservative treatment as a valuable therapeutic option in hemodynamically stable patients, particularly in resource‐constrained settings with limited access to endovascular embolization techniques.

## Graphical abstract

Post‐contrast axial (A) and sagittal (B) chest CT images (soft tissue window) showing an aneurysmal dilatation (red arrow) arising from the interloper branch of the left pulmonary artery (yellow arrow).

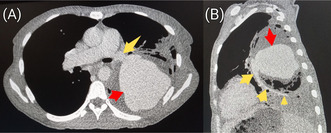

**Abbreviations**
AP: anteroposterior
CC: craniocaudal
CT: computed tomography
PA: posteroanterior
TB: tuberculosis
TRV: transverse

## 1 Introduction

Tuberculosis (TB) is an infectious disease caused by *Mycobacterium tuberculosis* and it frequently affects the lungs. In 2022, TB was the second leading infectious cause of mortality and was responsible for about 1.30 million deaths globally. An estimated 7.5 million new cases of TB were detected worldwide in 2022 [1]. In Ghana, the incidence of TB was reported to be 136 cases per 100,000 population in 2021 [2].

Delayed diagnosis and initiation of anti‐tubercular treatment substantially increase the risk of complications and mortality as well as transmission of the disease among the general population [3]. Pulmonary complications of TB include pleural effusion, empyema, bronchiectasis, spontaneous pneumothorax, chronic pulmonary aspergillosis, broncholithiasis, and tracheobronchial stenosis [4]. The actual burden of post‐TB lung disease in sub‐Saharan Africa is unknown due to the scarcity of data [5]. A review of the medical records of patients seeking care at a hospital in Uganda indicated that 30% of the attendees had post‐TB lung disease [6]. Rarely, patients who have cavitary pulmonary TB may develop Rasmussen aneurysm which is a pseudoaneurysm of a branch of the pulmonary artery within or adjacent to a tuberculous cavity [7, 8, 9]. The underlying pathologic process is characterized by progressive weakening of the arterial wall due to the infiltration of granulation tissue into the tunica adventitia and media. This is eventually replaced by fibrin, resulting in thinning of the vessel wall, pseudoaneurysmal dilatation, and subsequent rupture with hemoptysis, which can be massive and life‐threatening [10, 11]. Massive hemoptysis from ruptured Rasmussen aneurysm is associated with a mortality rate ranging from 5% to 25% [12]. Death is mostly due to aspiration and asphyxia from the intrapulmonary hemorrhage [13]. It affects about 1 out of 14,000 people (0.007%) [9, 14]. In sub‐Saharan Africa however, there is limited data regarding this rare entity. The lack of awareness, coupled with the rarity of the condition, may have contributed to the underdiagnosis of Rasmussen aneurysm in TB‐endemic areas like Ghana.

Rasmussen aneurysm is a nearly forgotten complication of pulmonary TB with few cases reported in the literature [15]. In this article, we discuss the case of a 43‐year‐old Ghanaian woman who had a giant‐sized Rasmussen aneurysm and was treated conservatively with no recurrence of hemoptysis.

## 2 Case History and Examination

A 43‐year‐old Ghanaian woman and a known diabetic with no previous history of pulmonary TB presented to our hospital with a 1‐month history of cough productive of whitish sputum. This was associated with intermittent low grade fever, drenching night sweats, and about 7.0 kg weight loss. Two weeks prior to presentation, she had progressively worsening hemoptysis (approximately 50–100 mL per day) along with chest pain, breathlessness on exertion, fatigue, palpitations, and dizziness. The hemoptysis was not preceded by any trauma to the chest. She was on premixed insulin (15 units mane and 10 units nocte), but not on any antiplatelet or anticoagulant. She denied exposure to anyone known or suspected to have pulmonary TB. She was a non‐alcoholic who neither smoked cigarettes nor used illicit drugs.

On physical examination, she was chronically ill‐looking, cachectic (weight = 42.0 kg), mildly febrile (37.5°C), anicteric, pale but not dyspneic. There was no peripheral or central cyanosis, finger clubbing, lymphadenopathy, or oral thrush. Her oxygen saturation was 94% on room air. She had a respiratory rate of 18 cycles per minute. Air entry was reduced bilaterally in the middle lung zones with bronchial breath sounds and coarse crackles on chest auscultation. She was tachycardic with a pulse rate of 104 beats per minute and had a blood pressure of 100/61 mmHg. Examination of the other systems was unremarkable.

## 3 Investigations

**FIGURE 1:**
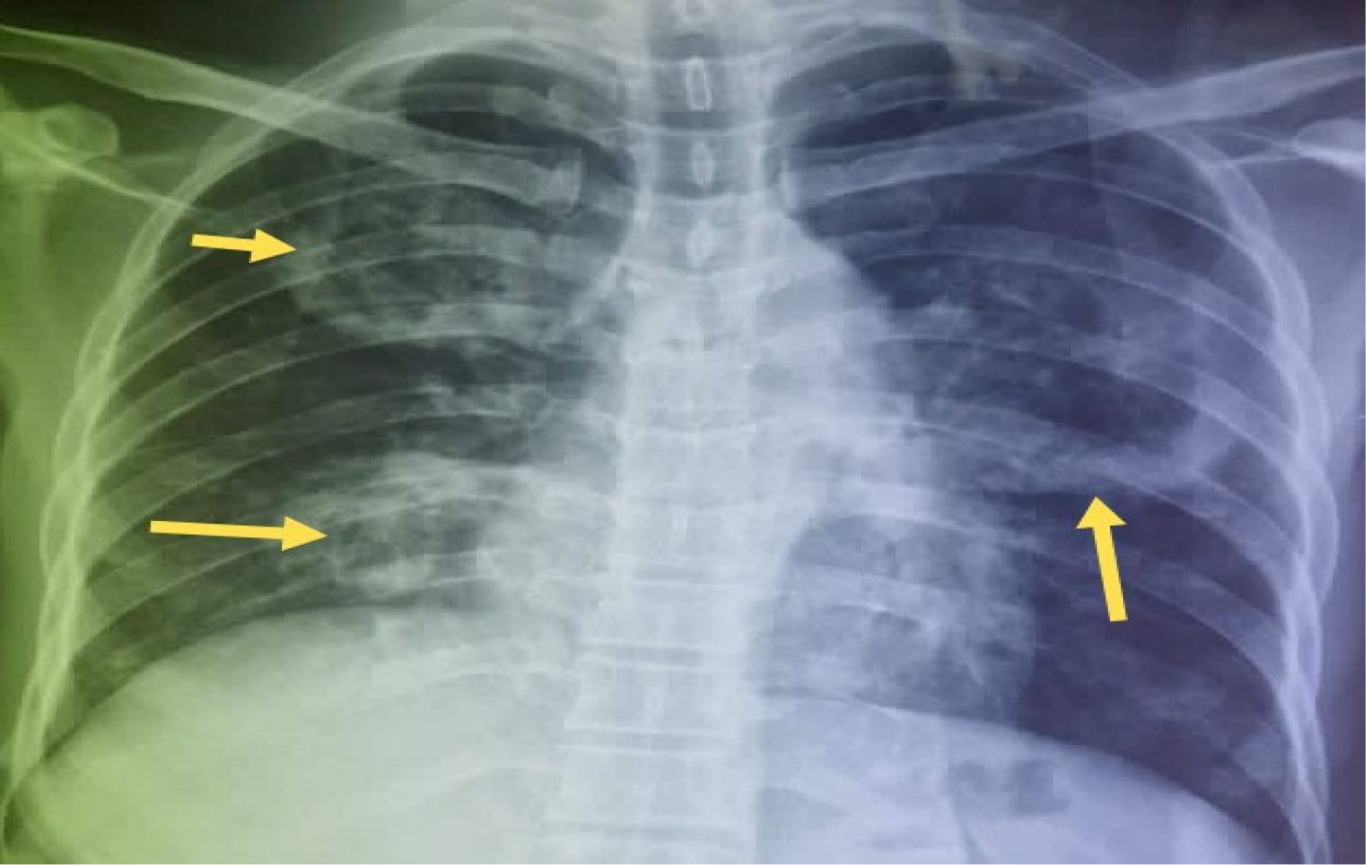
Posteroanterior (PA) chest radiograph showing cavitary lesions (yellow arrow) in the right and left lungs.

**FIGURE 2:**
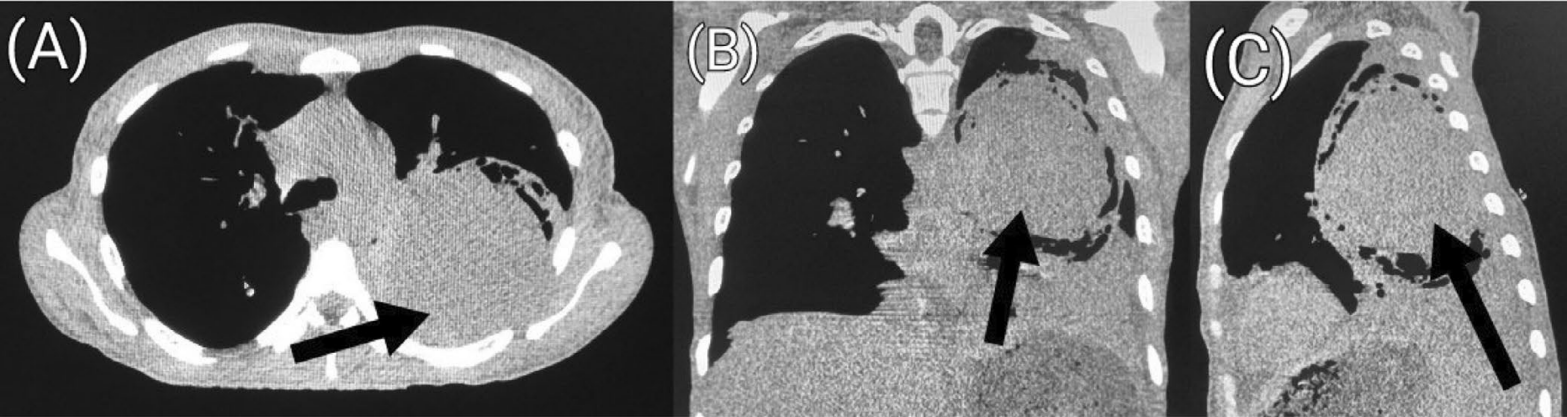
Pre‐contrast axial (A), coronal (B), and sagittal (C) CT scan images of the chest in soft tissue window demonstrating a large cavity (black arrow) in the upper lobe of the left lung.

**FIGURE 3:**
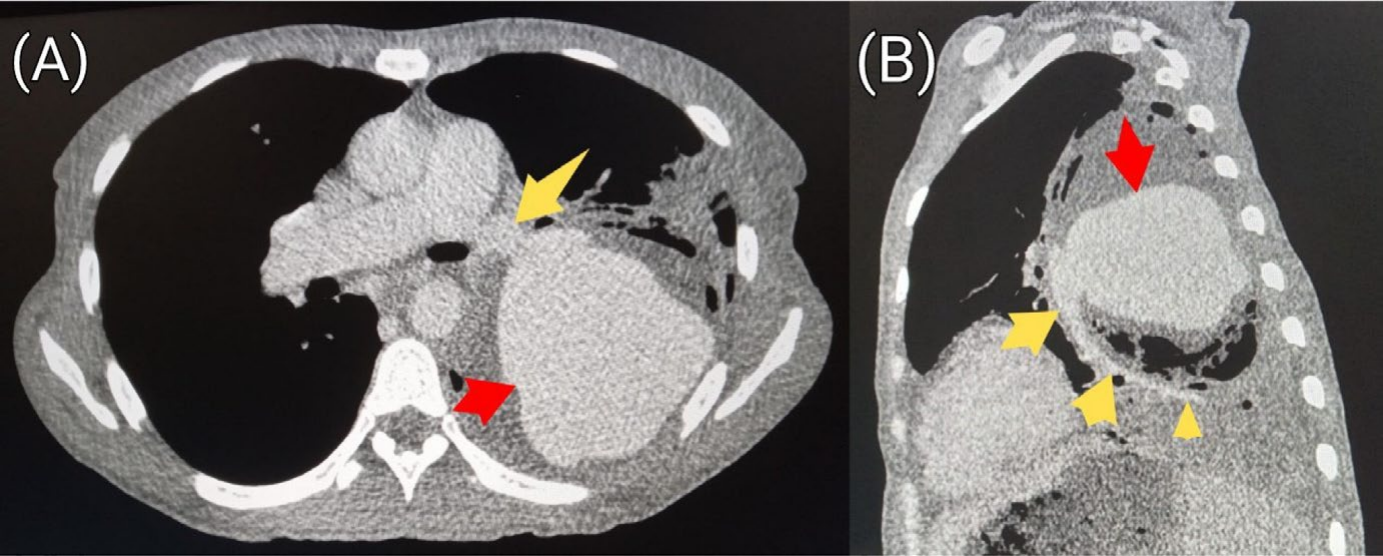
Post‐contrast axial (A) and sagittal (B) chest CT images (soft tissue window) showing an aneurysmal dilatation (red arrow) arising from the interlobar branch of the left pulmonary artery (yellow arrow).

**FIGURE 4:**
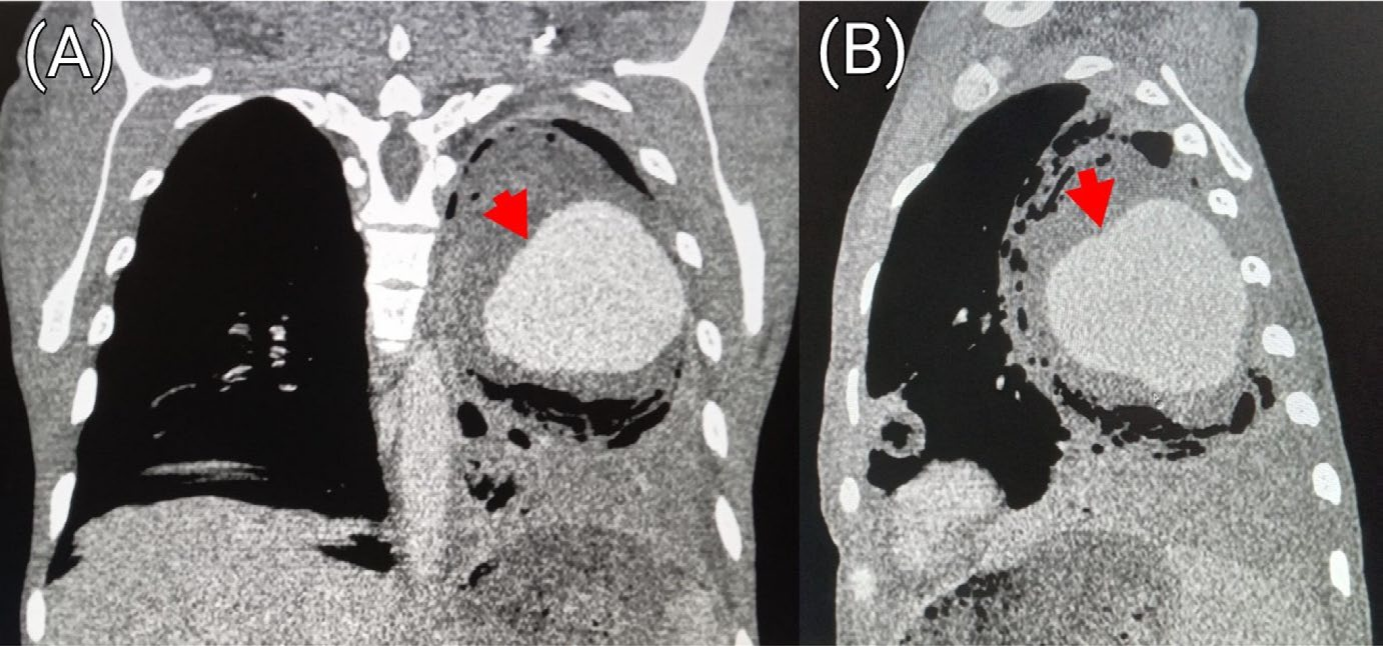
Contrasted coronal (A) and sagittal (B) sections of the chest in soft tissue window showing an aneurysmal dilatation (red arrow) with surrounding hematoma.

**FIGURE 5:**
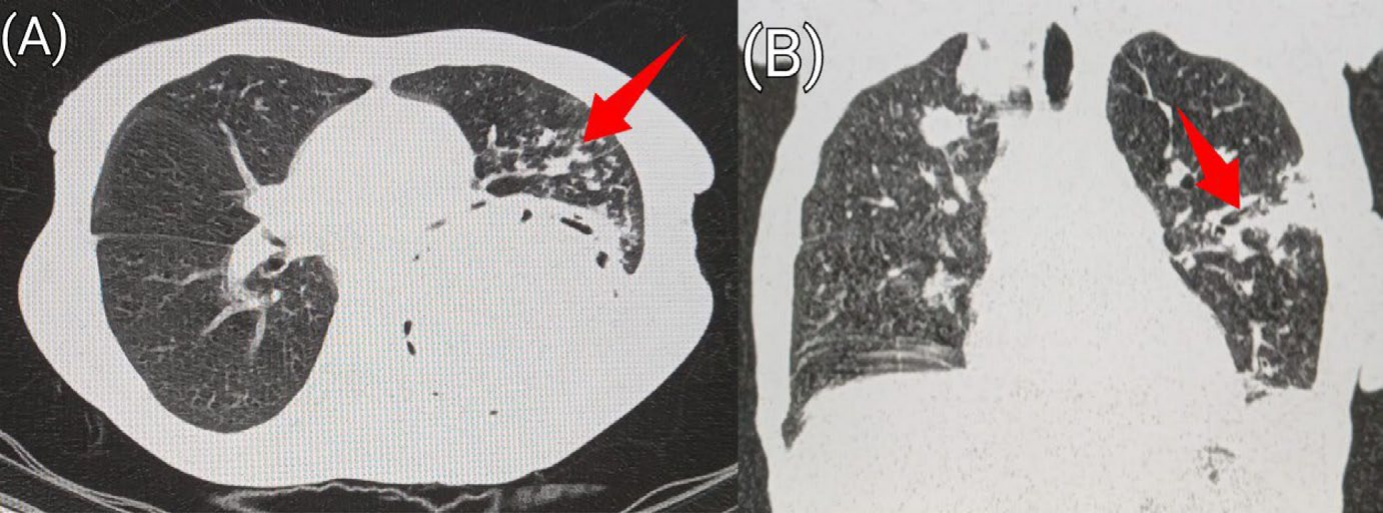
Lung window of pre‐contrast axial (A) and sagittal (B) CT sections of the chest showing fibrotic changes with interstitial thickening (red arrow) in the left lower lobe.

**FIGURE 6:**
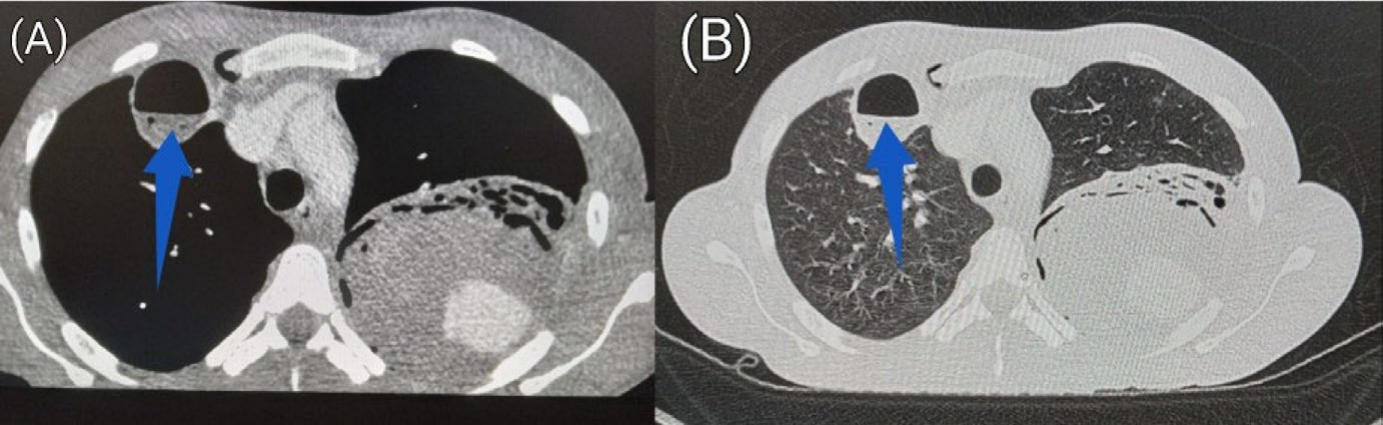
Post‐contrast axial CT sections of the chest in soft tissue (A) and lung (B) window demonstrating a small cavity (blue arrow) with air‐fluid level in the right upper lobe.

**FIGURE 7:**
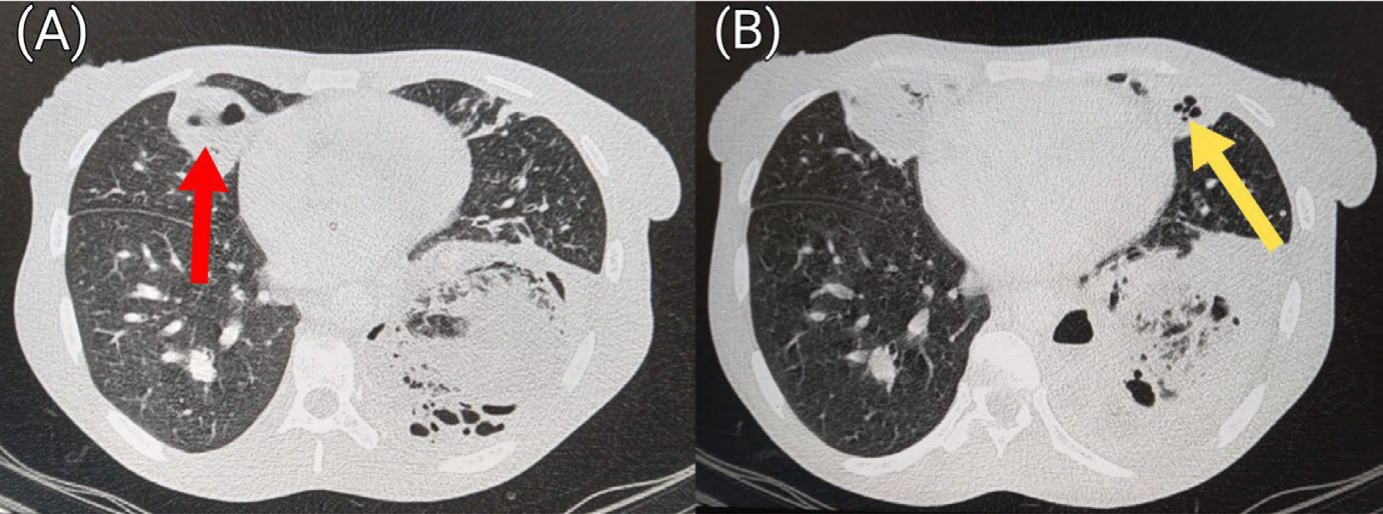
Pre‐contrast axial sections of the chest in lung window showing a small cavity with air‐fluid level in the right middle lobe (red arrow in A) and another small cavity without air‐fluid level in the left upper lobe (yellow arrow in B).

Laboratory investigations revealed decreased hemoglobin concentration (7.9 g/dL; reference range: 11.0–16.0 g/dL) and elevated erythrocyte sedimentation rate (110 mm/h; reference range: 0–20 mm/h). Serological test for human immunodeficiency virus was negative. Her fasting blood glucose and glycated hemoglobin were 6.8 mmol/L (reference range < 7.0 mmol/L) and 6.2% (reference range < 6.5%) respectively. Liver and renal biochemistries were normal. Cartridge‐based nucleic acid amplification test (Xpert MTB/RIF) for *M. tuberculosis* and sputum for acid‐fast bacilli yielded negative results. Chest radiograph showed cavitary lesions in the middle and lower zones of the right lung as well as the middle zone of the left lung (Figure 1). Pre‐contrast computed tomography (CT) scan of the chest showed a large cavity involving nearly the entire upper lobe of the left lung. The cavity measured 9.6 × 9.4 × 10.5 cm (transverse [TRV] × anteroposterior [AP] × craniocaudal [CC]) (Figure 2). Aneurysmal dilatation of the left interlobar pulmonary artery measuring 9.4 × 9.3 × 9.3 cm (TRV × AP × CC) with peripheral hematoma was visible within the cavity (Figures 3 and 4) on post‐contrast CT images of the chest. Additionally, there were fibrotic changes with interstitial thickening in the left lower lobe (Figure 5). Also present were smaller thick‐walled cavities with air‐fluid levels in the apical and medial segments of the right upper and middle lobes respectively. The size of the right upper lobe cavity was 3.5 × 3.7 × 3.9 cm (TRV × AP × CC), while the one in the right middle lobe measured 4.8 × 4.3 × 3.9 cm (TRV × AP × CC) (Figures 6 and 7A). Another smaller cavity without air‐fluid level was seen in the superior lingular segment of the left upper lobe (Figure 7B).

## 4 Diagnosis and Treatment

A clinical diagnosis of cavitary pulmonary TB complicated by Rasmussen aneurysm was made after evaluation. She was given intravenous tranexamic acid 1 g stat, followed by 500 mg three times daily for 2 days. She was also transfused with 2 units of packed red blood cells. Subsequently, the amount of hemoptysis reduced to about 10 mL per day and completely resolved by the third day of admission. Her repeat hemoglobin concentration was 10.8 g/dL. The need for either endovascular embolization or surgical intervention to prevent the risk of recurrent life‐threatening hemoptysis was extensively discussed with the patient and her husband. They, however, indicated they were financially constrained and therefore could not afford endovascular embolization. The patient also declined surgical intervention. Under the circumstances, she was started on a 6‐month regimen of anti‐tubercular drugs (i.e., 2 months of rifampicin 450 mg daily, isoniazid 225 mg daily, pyrazinamide 1200 mg daily, and ethambutol 825 mg daily followed by 4 months of rifampicin 450 mg daily and isoniazid 225 mg daily) together with pyridoxine 50 mg daily to prevent isoniazid‐induced peripheral neuropathy.

## 5 Outcome and Follow‐Up

**FIGURE 8:**
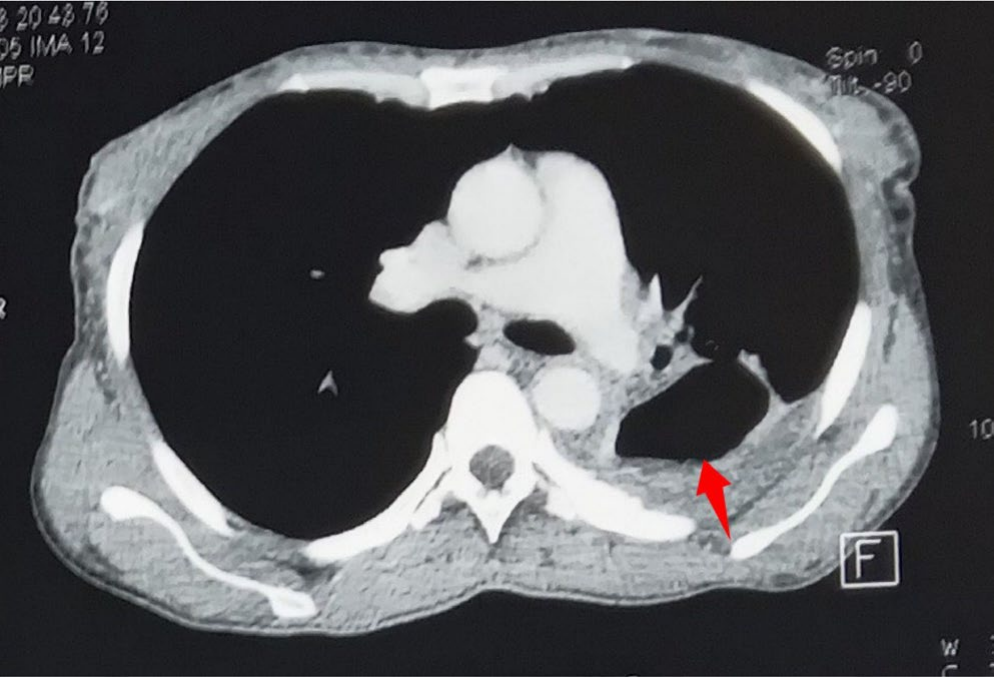
Post‐contrast axial CT scan image of the chest in soft tissue window showing a reduced left upper lobe cavity (red arrow). The Rasmussen aneurysm has completely resolved with normal appearance of the pulmonary vasculature.

**FIGURE 9:**
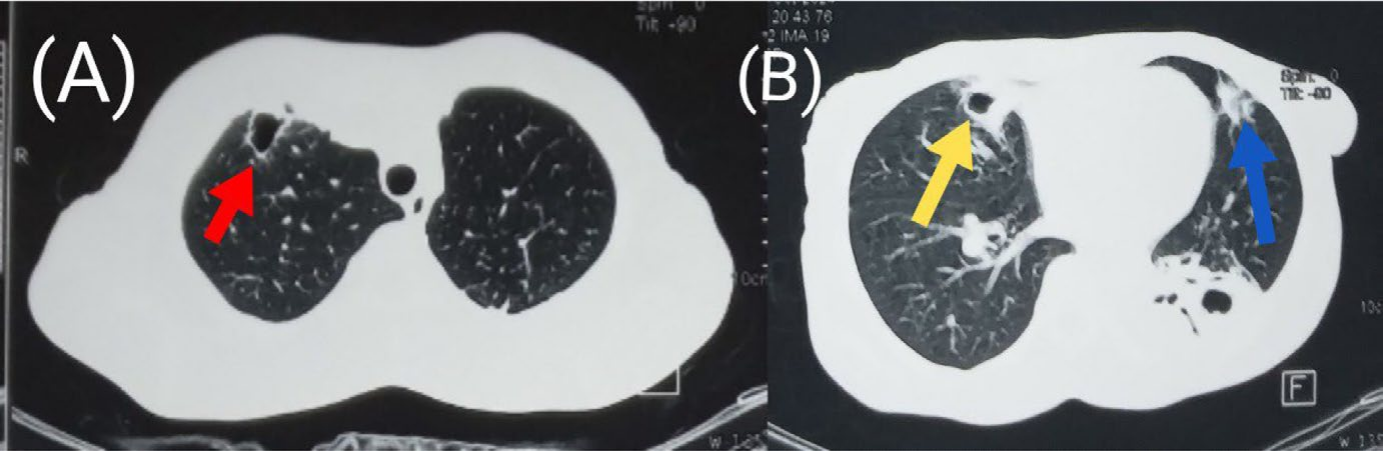
Pre‐contrast axial CT sections of the chest in lung window showing cavities with resolved air‐fluid levels in the right upper (red arrow in A) and middle (yellow arrow in B) lobes. Also noted is nearly complete resolution of the smaller cavity in the superior lingular segment of the left upper lobe (blue arrow in B).

At discharge 6 days after initiating anti‐tubercular treatment, the hemoptysis had completely resolved. At follow‐up after 5 months of taking anti‐tubercular drugs, there had been no recurrence of hemoptysis. She had gained weight (53.0 kg) with complete resolution of all her symptoms. Repeat CT imaging of the chest showed a reduced left upper lobe cavity which measured 5.6 × 4.2 × 8.3 cm (TRV × AP × CC). The Rasmussen aneurysm and the surrounding hematoma had completely resolved with a normal appearance of the entire pulmonary vasculature (Figure 8). The right upper and middle lobe cavities (measuring 1.2 × 1.6 × 1.7 cm [TRV × AP × CC] and 1.3 × 1.2 × 1.1 cm [TRV × AP × CC], respectively) had also reduced in size with disappearance of the air‐fluid levels (Figure 9). There was nearly complete resolution of the smaller cavity in the superior lingular segment of the left upper lobe (Figure 9). The need for strict medication adherence to ensure successful completion of the 6‐month anti‐tubercular regimen was reiterated.

## 6 Discussion

Hemoptysis refers to the coughing up or expectoration of blood originating from the tracheobronchial tree or lung parenchyma. It is an alarming and worrisome symptom that warrants careful clinical evaluation [16]. About 8% of individuals with pulmonary TB will experience hemoptysis at some point in their life [4]. In patients with pulmonary TB, massive hemoptysis often results from pathologies like bronchiectasis, aspergillomas, broncholiths, or vascular abnormalities. Among the vascular complications, bronchial arteries are the commonest source of hemorrhage while bleeding from the pulmonary arteries accounts for < 10% of hemoptysis [7, 17].

Rasmussen aneurysm is an inflammatory pseudoaneurysmal dilatation of a branch of the pulmonary artery that occurs within or adjacent to a tuberculous cavity [7, 8]. It is often located peripherally and beyond the branches of the main pulmonary arteries [10]. It is an uncommon vascular abnormality with a reported incidence of about 5% in patients with cavitary TB [18], and was first described in 1868 by Fritz Valdemar Rasmussen et al. [19] Although TB is endemic in Ghana, our report in addition to an earlier publication by Twum et al. [20] are the only two published case reports of Rasmussen aneurysm from this sub‐Saharan African country.

The patient presented with chronic productive cough, weight loss, drenching night sweats, fever, chest pain, fatigue, and hemoptysis. The significant auscultatory findings were bronchial breath sounds and coarse crackles. These clinical features are typically associated with active pulmonary TB [21]. Also, hemoptysis is the commonest manifestation in individuals with Rasmussen aneurysm [7, 8, 9]. Our patient's risk factor for pulmonary TB was diabetes mellitus. Several studies have demonstrated that the lifetime risk of developing TB increases by three‐fold in diabetics [22, 23, 24]. Diabetes mellitus suppresses the immune system, which makes diabetics susceptible to infection with *M. tuberculosis* and further enhances progression from latent to active disease [25].

A definitive diagnosis of active pulmonary TB requires a positive smear microscopy, culture, or nucleic acid amplification test for *M. tuberculosis* [26]. In the case of our patient, the results of cartridge‐based nucleic acid amplification test (Xpert MTB/RIF) and sputum smear microscopy were negative. However, her clinical features and chest imaging findings were highly suggestive of pulmonary TB, and were, therefore, clinically diagnosed as such. Cases of Rasmussen aneurysm in patients with clinically diagnosed pulmonary TB have been reported in Ghana [20] and Chile [27]. Globally, 63% of the 6.2 million individuals diagnosed with pulmonary TB in 2022 were bacteriologically confirmed, while the remaining number of cases were clinically diagnosed [1]. Also, a retrospective study conducted at the TB unit of a tertiary hospital in Ghana revealed that 18% of pulmonary TB cases were clinically diagnosed [28]. CT pulmonary angiography is the most preferred diagnostic tool for Rasmussen aneurysm [10]. In our patient, contrasted CT scan images of the chest classically showed an aneurysmal dilatation (9.4 × 9.3 × 9.3 cm) of the left interlobar pulmonary artery within a cavity in the upper lobe of the left lung. Rasmussen aneurysms frequently occur in the upper lobes on peripheral pulmonary arteries [13] such as the left interlobar pulmonary artery as observed in our case. A similarly sized pseudoaneurysm (9.5 × 7.0 cm) was detected in a 58‐year‐old man with pulmonary TB. Unlike our case, the aneurysm was located in the right lung and the sputum examination was positive for acid‐fast bacilli [29].

Currently, there are no studies that compare the conservative approach and other treatment modalities (i.e., endovascular embolization and surgical intervention). Nonetheless, endovascular embolization using gelfoam, coils, or plugs is supported by most case reports as the therapeutic intervention of choice because it is associated with significantly low morbidity and mortality [8, 11]. Several small studies have demonstrated endovascular embolization to be an effective treatment modality for pulmonary artery pseudoaneurysms including Rasmussen aneurysms [13, 30, 31]. Surgical interventions like lobectomy and pneumonectomy are indicated when endovascular embolization fails [8, 14]. In Ghana and many other low‐resource settings, endovascular embolization services are not readily available. These procedures are often expensive and barely affordable for most patients [32]. Our patient could not afford endovascular embolization and she also declined surgical intervention. As a result, we were constrained to treat her conservatively with anti‐tubercular drugs. Her hemodynamic stability at presentation coupled with her favorable clinical response to the administration of hemostatic agent (tranexamic acid) and blood transfusion, contributed to the successful conservative management. There have been few reports of successful conservative treatment of Rasmussen aneurysms. A 9‐year‐old boy with a large Rasmussen aneurysm measuring 3.8 × 5.0 × 5.7 cm was treated conservatively. Follow‐up imaging of the chest after 2 months showed a reduction in the size of the aneurysm with no recurrence of hemoptysis [8]. Two patients who tested positive for *M. tuberculosis* were subsequently found to have Rasmussen aneurysms on angiographic imaging of the chest. They were both treated with anti‐tubercular drugs for 6 months without further episodes of hemoptysis [14]. Twum et al. also reported the case of a 44‐year‐old Ghanaian with clinically diagnosed cavitary pulmonary TB. Chest CT scan showed a large Rasmussen aneurysm, which measured 7.0 × 5.8 × 7.0 cm. The patient was started on a 6‐month regimen of anti‐tubercular drugs. There was complete resolution of the aneurysm on follow‐up CT imaging of the chest performed 18 months after the initial presentation [20]. Another patient with bacteriologically confirmed pulmonary TB and a Rasmussen aneurysm measuring 2.3 × 2.6 cm was treated conservatively with anti‐tubercular drugs for 6 months. CT pulmonary angiography performed after 3 weeks revealed complete resolution of the pseudoaneurysm [33].

Although conservative management with close monitoring may be a valuable therapeutic option in carefully selected individuals with stable hemodynamics, particularly in resource‐deprived countries, giant‐sized Rasmussen aneurysms such as the one detected in our patient pose a significant risk of delayed rupture with life‐threatening hemoptysis due to the friable nature of the pseudoaneurysm. Other factors associated with the rupture of pulmonary artery pseudoaneurysms include large pulmonary cavitation, massive hemoptysis, and elevated mean pulmonary artery pressure [34]. Again, conservatively treated giant‐sized Rasmussen aneurysms may recur. Therefore, patients presenting with large Rasmussen aneurysms should be offered endovascular embolization or surgical intervention when feasible.

## 7 Conclusion

Rasmussen aneurysm is an uncommon complication of pulmonary TB. It should be considered as a differential diagnosis in patients with cavitary TB presenting with hemoptysis. CT pulmonary angiography is the gold standard diagnostic tool for this rare vascular condition. Endovascular embolization is the preferred therapeutic intervention. In resource‐constrained settings, conservative treatment with anti‐tubercular drugs is a valuable therapeutic option worth considering in hemodynamically stable patients.

## Author Contributions

**Prosper Adjei:** conceptualization, data curation, investigation, writing – original draft, writing – review and editing. **Edward Ebo Ocran:** investigation. **Jerry Kevin Boafo Osei‐Alomele:** data curation. **Belynda Abiti:** data curation.

## Ethics Statement

The authors have nothing to report.

## Consent

Written informed consent was obtained from the patient to publish this report in accordance with the journal's patient consent policy.

## Conflicts of Interest

The authors declare no conflicts of interest.

## Data Availability

The authors have nothing to report.
